# Different Trends in Excess Mortality in a Central European Country Compared to Main European Regions in the Year of the COVID-19 Pandemic (2020): a Hungarian Analysis

**DOI:** 10.3389/pore.2021.1609774

**Published:** 2021-04-13

**Authors:** Krisztina Bogos, Zoltan Kiss, Anna Kerpel Fronius, Gabriella Temesi, Jenő Elek, Ildikó Madurka, Zsuzsanna Cselkó, Péter Csányi, Zsolt Abonyi-Tóth, György Rokszin, Zsófia Barcza, Judit Moldvay

**Affiliations:** ^1^ National Korányi Institute of Pulmonology, Budapest, Hungary; ^2^ 2nd Department of Medicine and Nephrological Center, Faculty of Medicine, University of Pécs, Pécs, Hungary; ^3^ RxTarget Ltd., Szolnok, Hungary; ^4^ Department of Biomathematics and Informatics, University of Veterinary Medicine, Budapest, Hungary; ^5^ Syntesia Medical Communications Ltd., Budapest, Hungary; ^6^ 1st Department of Pulmonology, National Korányi Institute of Pulmonology, Semmelweis University, Budapest, Hungary; ^7^ 2nd Department of Pathology, MTA-SE NAP, Brain Metastasis Research Group, Hungarian Academy of Sciences, Semmelweis University, Budapest, Hungary

**Keywords:** Hungary, covid-19 pandemic, excess mortality, cumulative death, age-standardized mortality rate

## Abstract

**Objective:** This study examined cumulative excess mortality in European countries in the year of the Covid-19 pandemic and characterized the dynamics of the pandemic in different countries, focusing on Hungary and the Central and Eastern European region.

**Methods:** Age-standardized cumulative excess mortality was calculated based on weekly mortality data from the EUROSTAT database, and was compared between 2020 and the 2016–2019 reference period in European countries.

**Results:** Cumulate weekly excess mortality in Hungary was in the negative range until week 44. By week 52, it reached 9,998 excess deaths, corresponding to 7.73% cumulative excess mortality vs. 2016–2019 (*p*-value = 0.030 vs. 2016–2019). In Q1, only Spain and Italy reported excess mortality compared to the reference period. Significant increases in excess mortality were detected between weeks 13 and 26 in Spain, United Kingdom, Belgium, Netherland and Sweden. Romania and Portugal showed the largest increases in age-standardized cumulative excess mortality in the Q3. The majority of Central and Eastern European countries experienced an outstandingly high impact of the pandemic in Q4 in terms of excess deaths. Hungary ranked 11th in cumulative excess mortality based on the latest available data of from the EUROSTAT database.

**Conclusion:** Hungary experienced a mortality deficit in the first half of 2020 compared to previous years, which was followed by an increase in mortality during the second wave of the COVID-19 pandemic, reaching 7.7% cumulative excess mortality by the end of 2020. The excess was lower than in neighboring countries with similar dynamics of the pandemic.

## Introduction

On December 31, 2019, China reported a cluster of pneumonia cases of unknown etiology from the city of Wuhan which were later identified as the first cases of coronavirus disease 2019 (Covid-19) caused by the novel coronavirus termed severe acute respiratory syndrome coronavirus 2 (SARS-CoV-2) [1]. The first European case was reported from France on January 24, 2020 [2] The first wave of the coronavirus outbreak reached Europe in early spring 2020 and was soon acknowledged as a global pandemic by the WHO on March 11, 2020 [3]. By the end of July 2020, Europe had more than three million confirmed cases of Covid-19, with over 200,000 deaths [4, 5].

The impact of the COVID-19 pandemic was dissimilar in different European countries and the peak of the outbreak varied greatly across the region. During the first wave, several Western and Southern European countries were facing rapidly growing numbers of infections and deaths in the spring of 2020, with Spain and Italy being the epicenters of the pandemic in Europe with the highest number of confirmed cases and the highest number of Covid-19 related deaths, respectively [6]. In contrast, infections and deaths due to Covid-19 remained relatively low in other countries as a result of timely restrictions and the implementation of social distancing measures [7]. During the summer period, the infection continued to spread at a lower intensity, which led to several countries easing their restrictions and re-opening their borders. Subsequently, most European countries were hit by a second wave of the pandemic in early autumn 2020, which in many countries proved to be more severe than the first wave. Finally, some countries such as the United States, Japan and Malaysia are already experiencing a third wave of the pandemic [8, 9]. Interestingly, in Europe, the first wave of the pandemic primarily affected older populations, whereas the second wave started with a rapid spread among younger generations and eventually spread to the elderly [10]. The varying dynamics and changing magnitude in different countries may partly be attributed to different approaches to handling the first wave. By introducing very strict prevention measures early on, Central and Eastern European countries in general managed to avoid a great surge in the number of infections and deaths in spring 2020. In contrast, countries delaying the implementation of strict measures or following a herd-immunity approach such as Sweden and the United Kingdom (United Kingdom) experienced higher amplitudes of the first wave [7].

Excess mortality is a comprehensive measure of the total impact of the Covid-19 pandemic on deaths as it includes Covid-19 deaths that were not correctly diagnosed and reported as well as deaths from other causes that are attributable to the overall crisis conditions [11]. The Covid-19 pandemic has been associated with substantial excess mortality both worldwide and in Europe [12]. Excess mortality statistics from EU member countries clearly show significant increases in excess deaths compared to the baseline period of 2016–2019, with peaks observed in March–April 2020 and August–September–October 2020 [13].

Hungary was among the first to implement very strict preventive measures in spring 2020 including a nationwide lockdown starting from March 28, 2020, including reserved shopping hours for seniors, transition to home office in most companies and to online education at all levels, from elementary schools through universities and colleges In summer due to low infection rates the lockdown was eased, and by autumn elementary school education returned to face-to-face classes, while higher education remained on-line. As a result, the country experienced a relatively mild first wave in terms of COVID-19-related deaths compared to Western and Southern European countries, which was followed by a sharp increase in excess mortality during the second, autumn wave of the pandemic [14].

Excess mortality data reported from different countries as well as cross-country comparisons should be examined and interpreted in view of the above differences in the dynamics of the pandemic, and preferably for the whole period of the COVID-19 outbreak. Therefore, the primary aim of this study was to examine Hungarian standardized weekly excess mortality data for the whole year of 2020 compared to the average of the preceding 4 years 2016–2019 at weeks 26, 39, 51, and 52. Furthermore, we also aimed to compare Hungarian excess mortality and COVID-19 related mortality with those reported from other European countries, and to provide possible explanations for certain differences observed in the dynamics of the pandemic. As annual data are not yet universally available, excess mortality was calculated based on the latest available weekly mortality data.

## Methods

Our study was based on data from the European Statistical Office (EUROSTAT) which provides statistical data on the European Union (EU), the Euro area, EU member countries [13]. Weekly mortality data for 2016–2019 were available for all countries reporting to the EUROSTAT database, and served as a basis for excess mortality calculations for 2020. For presenting the impact of Covid-19 pandemic in 2020 for Hungary, average weekly mortality and average cumulative mortality were calculated for the 2016–2019 period. For Hungary, 95% confidence intervals (CI) and 95% prediction intervals were calculated based on the reference period, assuming normal distribution. Data from 2020 were compared to averages from the 2016–2019 period. Crude mortality was calculated as persons/week or persons/year-to-date in the cumulative version. The following age groups were created for our calculations: 0–34; 35–39; 40–44; 45–49; 50–54; 55–59; 60–64; 65–69; 70–74; 75–79; 80–84; 85 + years. For cross country comparison, crude mortality data of European countries were standardized for 100,000 persons. In 2016–2019, the population of January 1 of each year was used for standardization, whereas for 2020 we also used the population of January 1, 2019, as population data for 2020 were not yet available for all countries in the EUROSTAT database. Mortality data from all countries were based on the European Standard Population 2013 to allow for between-country comparisons (using 40,000; 7,000; 7,000; 7,000; 7,000; 6,500; 6,000; 5,500; 5,000; 4,000; 2,500; 2,500 people for age cohorts respectively). Cumulative excess mortality and age-standardized excess mortality were also calculated for the following European regions: Scandinavian countries (Denmark, Finland, Sweden, Norway, Iceland); Baltic countries (Estonia, Latvia, Lithuania); Benelux countries (Belgium, the Netherlands, Luxembourg); Western EU countries (Austria, France, Germany, Liechtenstein, Switzerland, United Kingdom); Mediterranean EU countries (Cyprus, Greece, Italy, Malta, Portugal, Spain); Balkan EU countries (Bulgaria, Croatia, Slovenia); Central-Eastern EU countries (the Czech Republic, Hungary, Poland, Romania, Slovakia) and V4 countries separately (the Czech Republic, Hungary, Poland, Slovakia).

We were using a prediction interval-based method. We calculated the probability that data from 2020 falls into the interval determined by data from last years as *p*-value. We were calculated the *p*-value from the Student’s t-distribution with n-1 degrees of freedom, because $\frac{X_{2020}-{\bar{X}}_{n}}{s_{n}\sqrt{1+1/n}}∼T_{n-1}$ where $X_{2020}$ is the standardized mortality in 2020, ${\bar{X}}_{n}$ is the mean of the standardized mortality in last years, $s_{n}$ is the estimated standard deviation, n=4 is the number of the preceding years. We were using the “R” statistical software v4.0.2 (2020-06-22).

## Results

**FIGURE 1:**
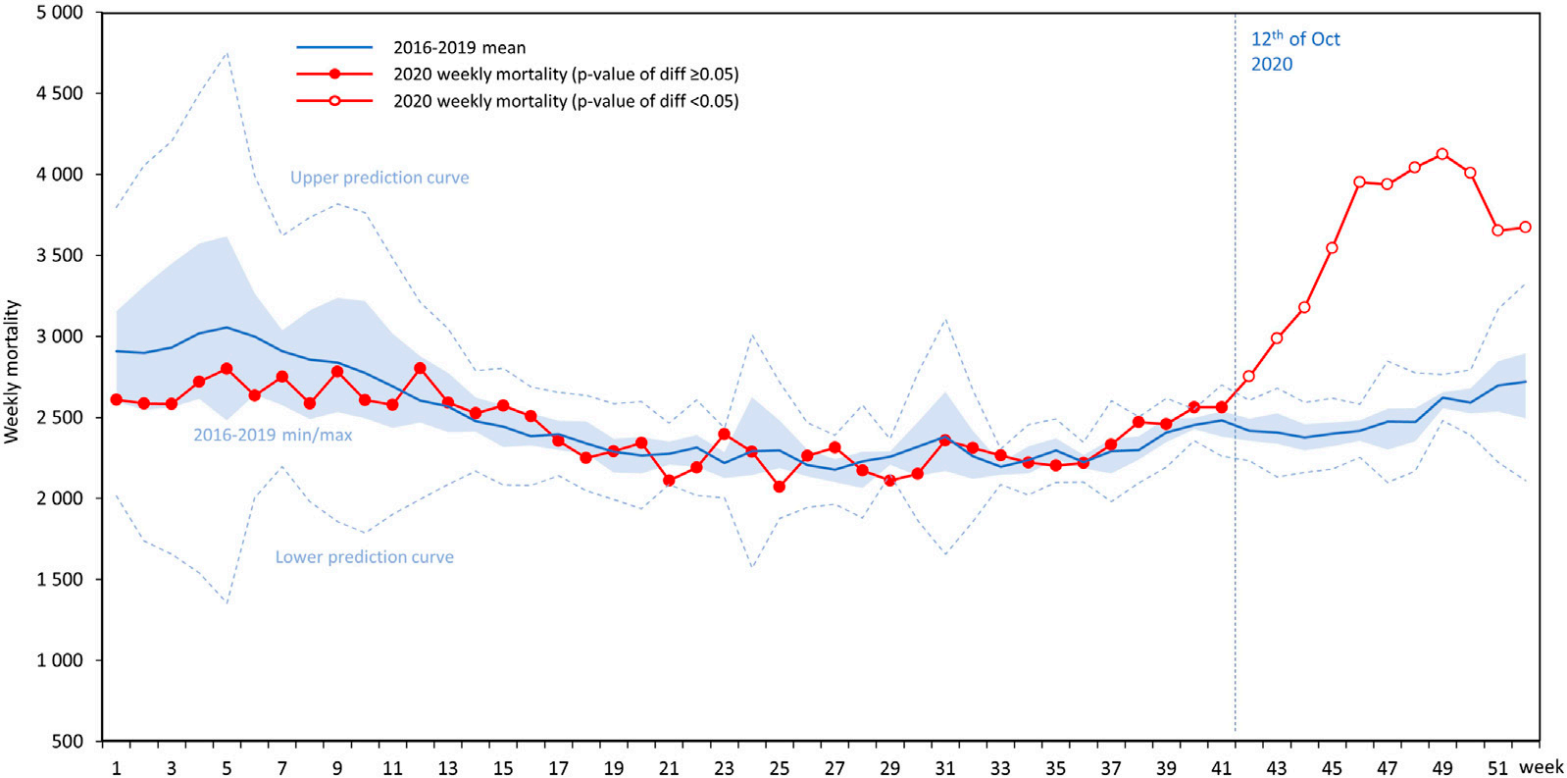
All-cause mortality in 2020 in Hungary by weeks compared to mean values from the period of 2016–2019 with 95% prediction intervals, together with the 2016–2019 lower and higher weekly mortality range (light blue range).

During the 4-year period of 2016–2019, mean weekly all-cause mortality varied between 3,054 and 2,178 in Hungary **(** Figure 1 **)**, with higher numbers in the early weeks of the year and in the fourth quarter (Q4, highest number: 3,616 deaths in week 5, 2017), and lower mid-year numbers (lowest number: 2,065 deaths, week 28, 2019).

In the first 11 weeks of 2020, weekly mortality was lower compared to the 2016–2019 average of the same period (difference was not significant Supplementary Table S1), while there were no relevant differences from week 12 to week 36. On the other hand, the number of weekly deaths exceeded the average of the preceding 4 years starting from week 37. Between weeks 42 and 52, significantly higher weekly mortality was recorded in Hungary than in the reference period of 2016–2019.

**FIGURE 2:**
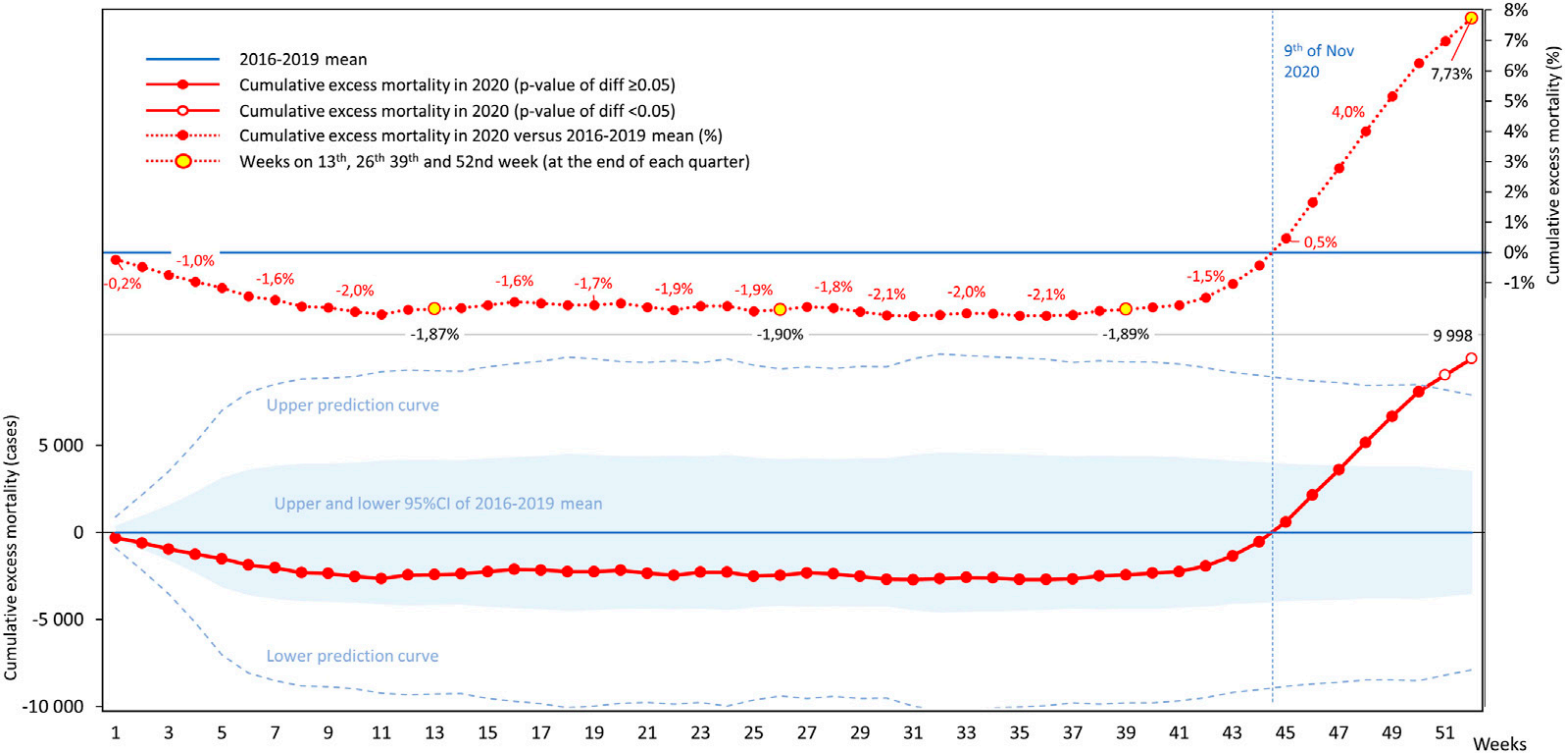
Cumulative all-cause excess mortality in Hungary by weeks in 2020 compared to averages from the reference period (2016–2019).

Cumulative all-cause excess mortality was lower until week 44 in 2020, than the mean cumulative excess mortality of the 2016–2019 period (Figure 2), corresponding to fewer Hungarian deaths from week 1 to 44 in 2020 than in the previous 4 years. Starting from week 44 (November 9, 2020), cumulative excess mortality increased to 9,998 deaths by week 52, exceeding the upper prediction curve in the last 2 weeks of 2020 (*p* = 0.010 in week 51, and *p* = 0.021 in week 52 for 2020 vs. 2016–2019). Excess mortality was - 1.9% in weeks 13, 26, and 39, corresponding to mortality deficit compared to the previous 4 years' average, while it increased to 7.73% by week 52 (Supplementary Table S1).

**FIGURE 3:**
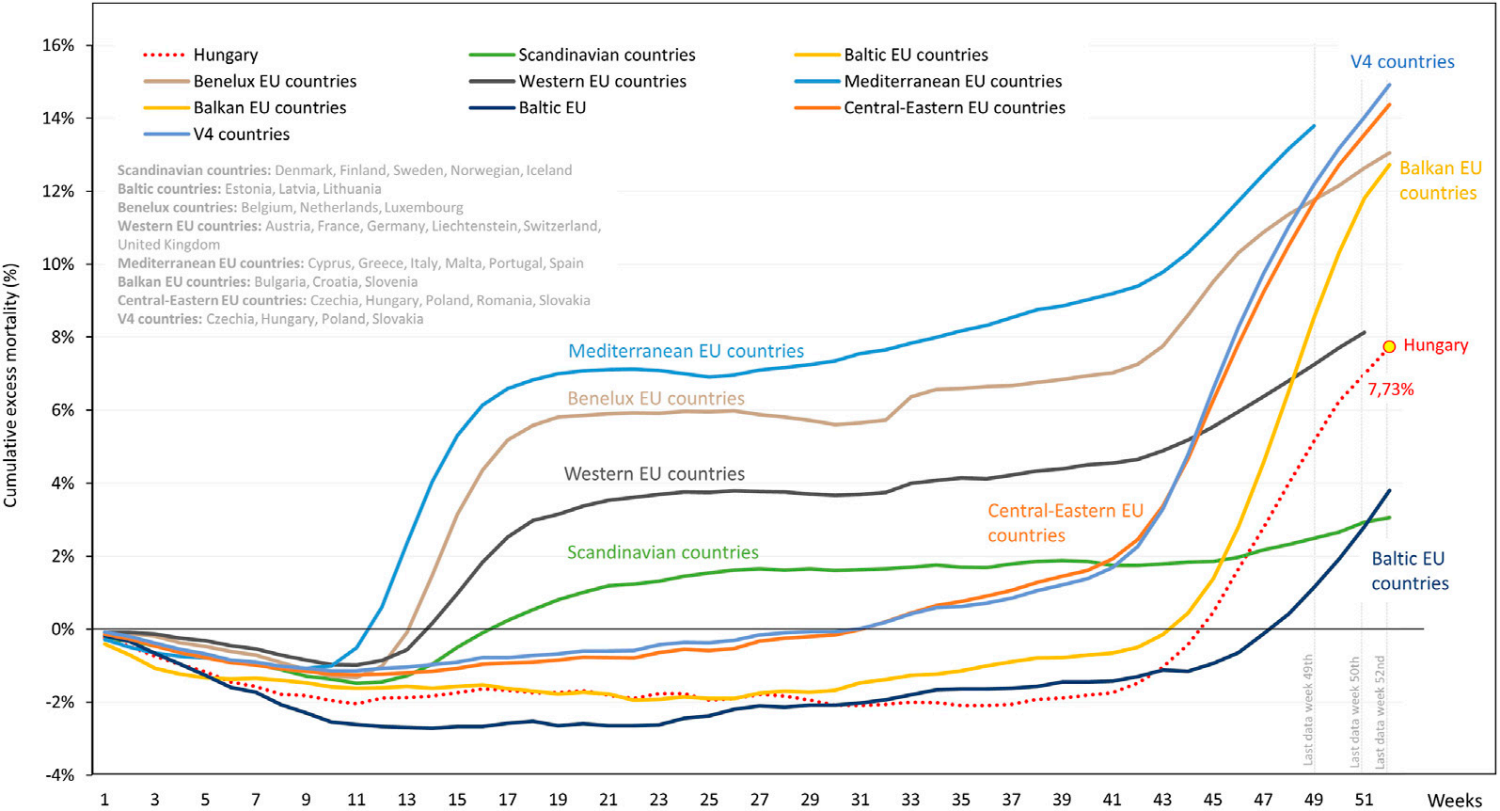
Weekly cumulative excess mortality in 2020 in Hungary compared to European regions. European regions are defined as Scandinavian countries for Denmark, Finland, Sweden, Norwegian, Iceland; Baltic countries for Estonia, Latvia, Lithuania; Benelux countries for Belgium, Netherlands, Luxembourg; Western EU countries for Austria, France, Germany, Liechtenstein, Switzerland, United Kingdom; Mediterranean EU countries for Cyprus, Greece, Italy, Malta, Portugal, Spain; Balkan EU countries for Bulgaria, Croatia, Slovenia; Central-Eastern EU countries for Czechia, Hungary, Poland, Romania, Slovakia; V4 countries for Czechia, Hungary, Poland, Slovakia.

Cumulative all-cause excess mortality exceeded 6% by week 16 in Mediterranean EU countries, almost reached 6% in Benelux countries, 4% in Western EU countries (plus Lichtenstein, United Kingdom and Switzerland) and 2% in Scandinavian countries by mid - 2020 (Figure 3 and Supplementary Table S2). On the other hand, no excess mortality was detectable in Central and Eastern, Balkan, and Baltic EU countries including the V4 country group during this period. During the second half of the year, excess mortality exceeded 12% in Benelux, approximated 14% in Mediterranean, and 8% in Western EU countries. A rapid increase in excess mortality was observed in Central and Eastern, Balkan, and Baltic EU countries in Q4 2020.

**FIGURE 4:**
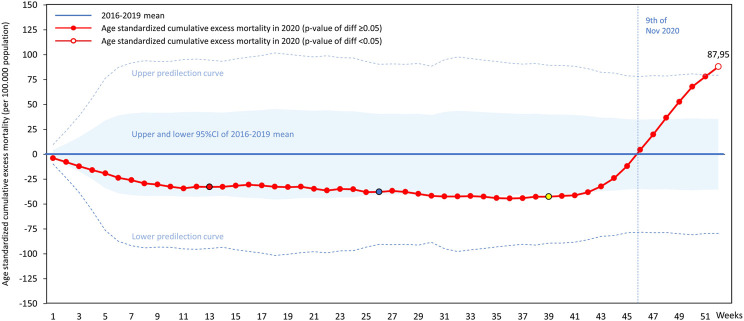
Hungarian age-standardized cumulative excess mortality in 2020 by weeks per 100,000 persons (colored weeks are reference points for Figure 6).

In Hungary, age-standardized cumulative excess mortality per 100,000 persons was also lower in the first 45 weeks of 2020 compared to 2016–2019 averages. The lowest value was detected in week 36 with - 44.35 deaths per 100,000 persons, which started to increase from the beginning of Q4 (week 40), exceeded the zero point on November 5, and reached 87.95 deaths per 100,000 persons in week 52. No significant differences were observed compared to the previous 4 years until week 52, after which age-standardized excess mortality significantly exceeded the upper predictive curve of the preceding 4-year period until the end of the year (Figure 4 and Supplementary Table S1).

**FIGURE 5:**
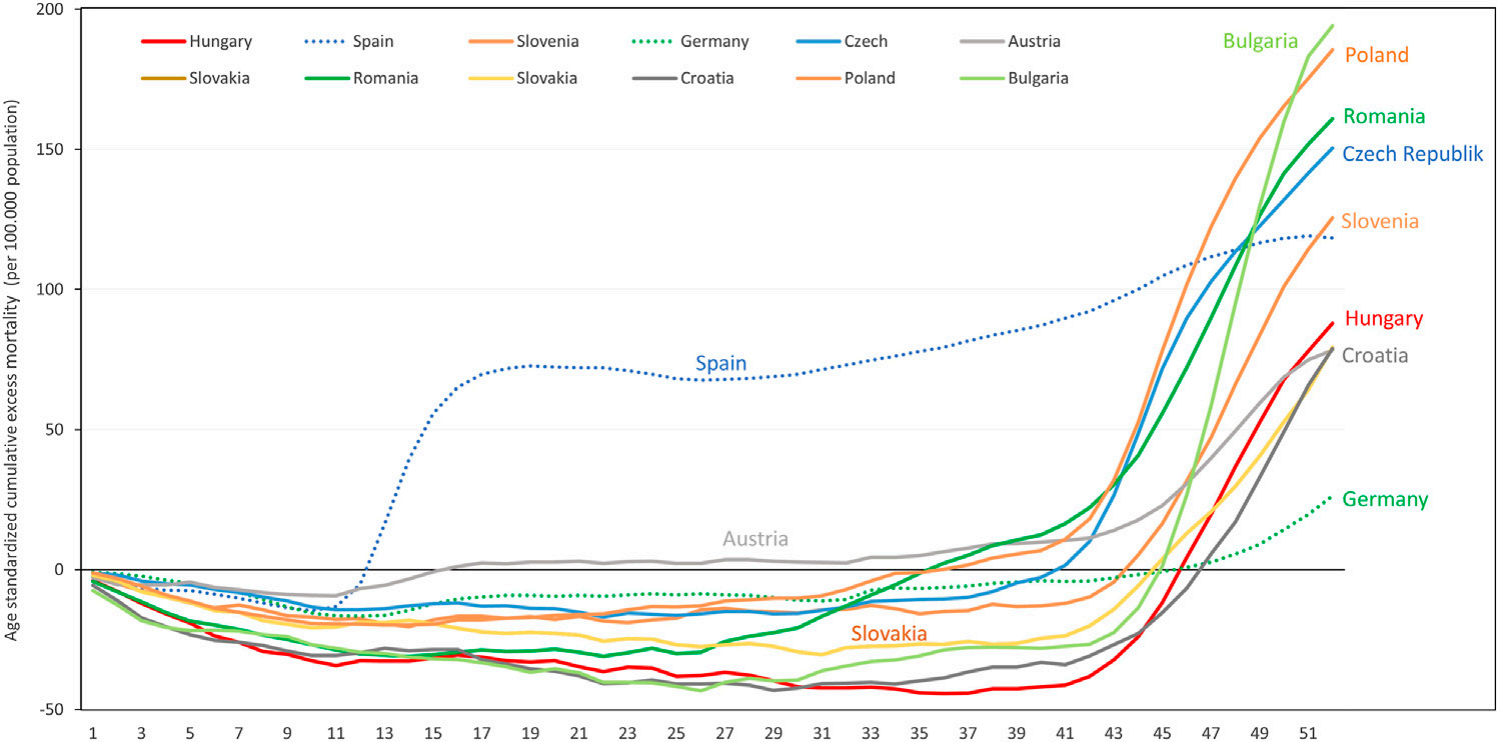
Age-standardized weekly cumulative excess mortality in 2020 in Hungary compared to selected, neighboring European countries (per 100,000 persons).

Figure 5 shows the age-standardized cumulative excess mortality curve of Hungary in 2020 compared with neighboring European countries (with Germany and Spain as reference). All countries in this region had mortality deficit in the first half of the year, with significant increases in excess mortality during the autumn-winter period of the pandemic. Hungary had substantially higher age-standardized excess mortality by week 52, than Germany, nearly the same as Austria, Slovakia and Croatia, but lower than the Czech Republic, Slovenia and Romania. Poland and Bulgaria accumulated more than twice as much excess mortality as Hungary (Supplementary Table S3).

**FIGURE 6:**
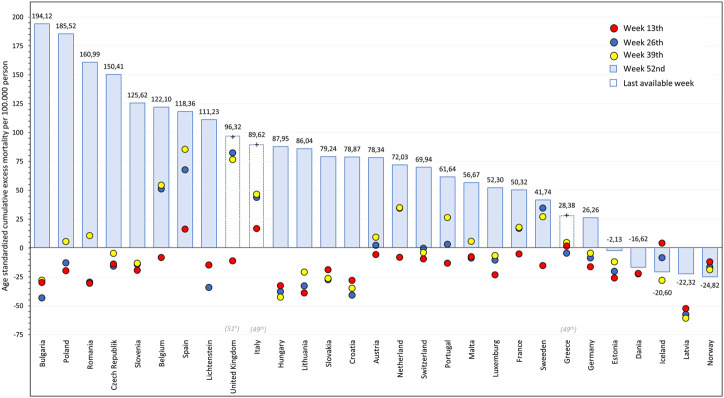
Age-standardized weekly cumulative excess mortality of European countries in 2020 at week 13th, 26th, 39th, and 52th (per 100,000 persons) (in case of United Kingdom, Italy and Greece, latest available week is highlighted in gray at countries name).

Figure 6 illustrates the dynamics of the pandemic by comparing age-standardized cumulative excess mortality of assessed European countries at the end of each quarter of 2020 (weeks 13, 26, 39, and 52), and highlighting the latest available weekly cumulative values from 44 separately for each country. There was no age-standardized excess mortality in EU countries in Q1 of 2020, except for Spain and Italy where 16.24 and 16.87 excess deaths per 100,000 persons were detected, respectively. In certain countries including Spain, the United Kingdom, Belgium, the Netherlands, and Sweden, significant increases in excess mortality were recorded in Q2 2020 (increases: 51.34; 93.60; 59.24; 42.53, and 49.74/100,000 persons, respectively). In Q3, only Romania and Portugal recorded more than 20 deaths per 100,000 persons, corresponding to 40.19 and 23.25/100,000 increases in age-standardized cumulative excess mortality. On the other hand, the COVID-19 pandemic had outstandingly high impact in certain Central Eastern European countries in Q4 2020 (Supplementary Table S3 and Supplementary Figure S1).

Bulgaria reported the highest increase in age-standardized, cumulative excess mortality (222.00 deaths), followed by Poland (180.02 deaths), Romania (150.39) and the Czech Republic (155.20 deaths), Slovenia (138.91 deaths), and Hungary (130.60 deaths) per 100,000 persons. At the end of 2020, Hungary reported 78.87 deaths per 100,000 persons, ranking 11th among countries with available EUROSTAT data with relevantly a lower value than Bulgaria, Poland, Spain, Belgium, the United Kingdom, the Czech Republic, Romania and Slovenia.

The number of recorded Covid-19 related deaths and total excess deaths, as well as the ratio of COVID-19 related mortality and total excess mortality in European countries and regions in 2020 are shown in (Supplementary Table S4). In most Central and Eastern European countries, Covid-19 related deaths accounted for less than two thirds of total excess deaths (Slovakia: 36.23%; Poland: 37.86%; Romania: 45.29%; Czech Republic: 65.54%). In Hungary, the number of cumulative excess deaths at week 52 was 9,998 and 9,047 reported COVID-19 related deaths occurred (90.49%), reflecting the mortality deficit observed in the first half of the year compared to previous years.

## Discussion

This analysis examined excess mortality in Hungary in 2020 compared to the preceding 4 years in view of the COVID-19 pandemic and compared findings to other European countries. The results show that despite the first wave of the COVID-19 pandemic, Hungary had lower all-cause mortality in the first half of 2020 compared to the average of the preceding 4 years. However, cumulative excess mortality increased to 7.7% by the end of 2020 compared to 2016–2019, which was due to the more rapid spread of the infection during the last quarter of 2020. Cumulative excess mortality was lower than that of most European countries with more severe first and second waves, and also lower compared to neighboring countries where the second wave resulted in more than 10% excess mortality compared to the preceding years.

Based on the findings of the current analysis, European countries can be classified based on the dynamics of excess mortality throughout the pandemic. Several Western, Benelux and Mediterranean European countries including Spain, Belgium, Italy, Sweden, France, Austria and the United Kingdom experienced high mortality toll with excess mortality during the first wave in spring 2020, which further increased during the second wave after a mid-year plateau. By the end of 2020 or the latest week with available mortality data, cumulative excess mortality exceeded 10% in most of these countries. Central and Eastern European and Balkan countries including Hungary, Poland, Romania, Slovenia, Bulgaria and the Czech Republic showed mortality deficit in the first half of the year. However, the second wave of the pandemic resulted in significant increase in cumulative excess mortality in these countries, mostly in the last quarter of 2020, which in Poland and Slovenia also exceeded 10%. Finally, no relevant excess mortality was found during 2020 in Scandinavian countries, except for Sweden, where excess mortality during the first wave was followed by a marginal mortality deficit in the third quarter, and cumulative excess mortality remained around 7% by the end of the year. This was also the situation in most Baltic countries, except for Lithuania.

The heterogeneous mortality effects of the Covid-19 pandemic may be explained by several factors. Substantial excess mortality during the first wave may be attributed to delays in restrictions or lenient policies and the lack of experience with Covid-19 treatment [15]. Limited hospital capacity, overwhelmed intensive care units and staff shortages may also have contributed to the excess mortality [16]. Furthermore, poor adherence to Covid-19 restrictions and social distancing measures as well as loosening restrictions for economic recovery in these countries may have played a part in the further increase of excess mortality during the second wave [17]. In addition, recent data from the United Kingdom suggest that a substantial proportion of excess mortality in the second half of 2020 may have derived from long-term complications of the first wave leading to hospital readmissions and deaths [18]. Finally, we cannot exclude the role of emerging new coronavirus variants in the excess mortality of the second half of 2020 [19].

The lack of excess mortality during the first half of 2020 compared to 2016–2019 in certain countries can most likely be attributed to the timely introduction of restrictions and social distancing measures in spring 2020 [20]. Hungary was among the countries that acted very early in terms of movement restrictions and lockdowns, and thus managed to keep the number of COVID-19 cases at such low levels that allowed for the identification and isolation of cases as well as efficient contact tracing [21, 22]. Restrictions and social distancing measures including the absence of major public events resulted in a less severe influenza season and reduced the spread of other infectious diseases such as pneumococcal pneumonia as well as the number of traffic accident fatalities [23]. Furthermore, the population in post-socialist Eastern European countries may have been more disciplined in respecting the instructions of self-isolation and confinement and took the coronavirus threat more seriously. Therefore, their populations were less severely affected by the first wave, than that of Western European countries, and not even the limited capacity of healthcare facilities resulted in an increase in mortality. The high-risk populations of these countries were spared during the first wave and were still alive when the second wave reached them, primarily spreading from the younger generations, which explains the sudden and significant mortality surge in the second half of 2020 observed in these countries [24, 25]. However, in some Baltic and Scandinavian countries, available cumulative mortality numbers did not show relevant excess mortality during 2020, most probably due to timely restrictions and higher healthcare expenditure per capita compared to other European countries [26].

While Hungary was ranked among firsts in the number of reported COVID-19 related death per 1 million inhabitants (based on Eurometer database first of Jan), there was no relevant difference between reported COVID-19 and total excess mortality (90.49% based on Supplementary Table S1). In contrast, certain neighboring countries as well as other European countries reported Covid-19 related mortality to total excess mortality ratios below 50%, hence, the COVID-19 related deaths per 1 million population ranking does not reflecting the real impact of the pandemic. A possible explanation for this result is the different methodology in COVID-19 related death reports as well as that a relevant proportion of patients may have died in their homes and hence were not registered in the system as COVID-19 related deaths. Furthermore, the pandemic may have indirect effects on mortality. The reduced number of hospital visits for fear of contracting the coronavirus or “bothering the doctor” and the postponement of screening services and surgical procedures due to the high demand for hospital beds and overwhelmed healthcare facilities may have led to otherwise avoidable deaths, especially in the case of cardiovascular conditions [27–29]. Long-term complications of COVID-19 may also account for a certain proportion of total excess mortality not classified as COVID-19 related.

Cumulative excess mortality is a general aspect about the impact of the pandemic which helps adjust for differences in COVID-19 related death reporting methodology and calculations. Relevant differences in the ratio of reported Covid-19 related mortality and total excess mortality revealed in our study confirm the benefit of excess mortality calculations for cross-country comparisons of the COVID-19 burden. Especially as some of the countries reports those whom died and had a positive COVID-19 test at the same period, some others report a death as COVID-19 related only if was the confirmed by clinical diagnosis, yet again others confirmed a death as COVID-19 related, if the patient did not have any other severe comorbidity.

In addition, in our excess mortality calculations for 2020, countries served as their own control groups for assessing scale of COVID-19 impact, adjusting for cross-country differences in mortality risks which derive from differences in health policies and health literacy over the past decades (as seen in Supplementary Table S5).

There are differences in the age distribution of European countries, and within-country changes in age distribution may also have occurred in 2020 compared to the previous years. Therefore, age-standardized excess mortality is a useful tool for comparing the impact of the pandemic on different countries and regions. Furthermore, the calculation of cumulative excess mortality allows for cross-country comparisons even if annual data are incomplete for certain countries. Early and standardized cross-country comparisons provide a reliable basis for the evaluation of the impact of restrictions and the response of healthcare systems for the COVID-19 pandemic.

It is important to consider that the reported numbers of weekly mortality may be re-validated over time, and later reports may change final results. There may be weeks of even months between death events and mortality data due to delays in reporting. Nevertheless, timely cross-country comparisons of the impact of the COVID-19 pandemic on mortality may aid governments in fast decision-making during challenging times. Finally, since EUROSTAT has not reported the age distribution of the population from 2020 for all European countries yet, hence our calculations for age standardization in 2020 were based on populations from 2019, which may have influenced the results in certain countries when 2020 population data will be available.

## Conclusion

During the first half of 2020, Hungary had a mortality deficit compared to the previous 4 years as a result of restrictions and social distancing measures implemented during the COVID-19 pandemic. Following the low amplitude of the first wave in spring 2020, a significant proportion of the population were infected during the second wave in autumn, which eventually resulted in a cumulative excess mortality of 7.7% by the end of 2020. However, the excess was lower than in neighboring countries with similar dynamics of the pandemic, and lower, than that of countries with more intense first and second waves.

The observed differences in the dynamics of the pandemic underline the importance of taking into account the whole year of 2020 instead of separate waves when making cross-country comparisons. As long as annual excess mortality data are not available, cumulative weekly or monthly excess mortality should be calculated for the analysis of COVID-19 waves in different countries. The calculation of cumulative excess mortality provides valuable insights into cross-country differences in the dynamics of the pandemic and may serve as a basis for evaluating the effectiveness and timeliness of restrictions and responses to this unprecedented global crisis.

## Data Availability

The original contributions presented in the study are included in the article/Supplementary Material, further inquiries can be directed to the corresponding author.
