# Supplementary figures and images for: Different Trends in Excess Mortality in a Central European Country Compared to Main European Regions in the Year of the COVID-19 Pandemic (2020): a Hungarian Analysis
Source: Pathol Oncol Res. 2021 Apr 13;27:1609774. doi: 10.3389/pore.2021.1609774 (PMC8262208; doi:10.3389/pore.2021.1609774)

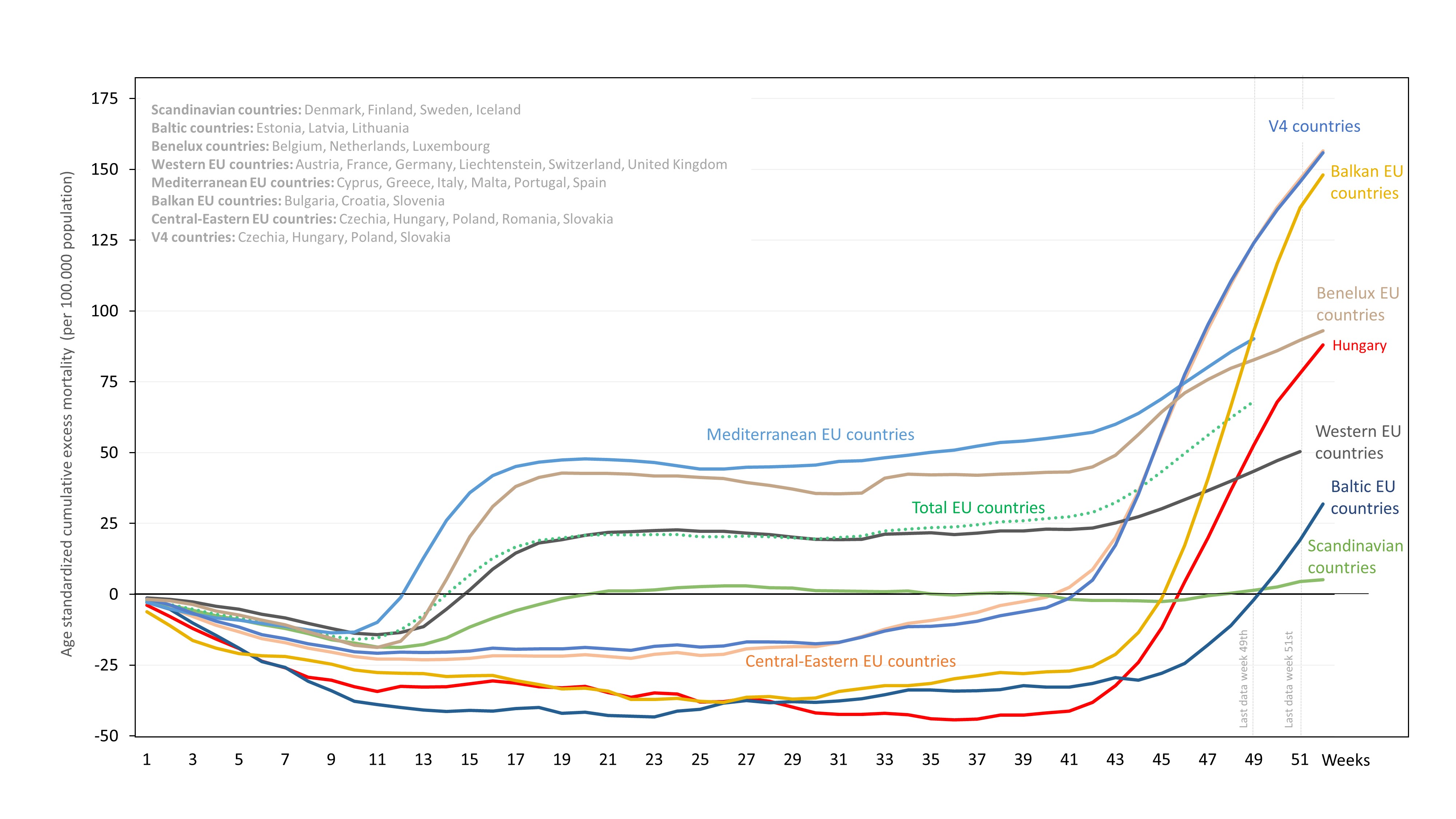

Supplement: Supplementary file 1 [file Image1.JPEG]
